# Leaders’ induced justice perceptions as mediator of the relation between participative leadership behaviors and team learning

**DOI:** 10.3389/fpsyg.2023.1244273

**Published:** 2023-11-27

**Authors:** Andres Raineri

**Affiliations:** School of Management, Pontificia Universidad Católica de Chile, Santiago, Chile

**Keywords:** team learning, participative leadership, justice climate, fairness theory, social exchange theory

## Abstract

**Introduction:**

In today’s complex and changing business environment organizations need to learn and adapt to emerging circumstances. Teams can be a preferred vehicle to facilitate solving challenges that require diverse perspectives and expertise, collaboration, and knowledge sharing among members. To support team learning, organizations need to understand and promote an appropriate environment that facilitates learning within teams. By drawing on Fairness Theory and Social Exchange Theory, this study explores the role of leader-induced justice perceptions as a mediator in the relationship of participative leadership and team learning.

**Methods:**

Using a split-half team survey methodology with a sample of 211 teams, the study analyzes the role of team justice climate as a mediation mechanism in the relationship between participative leadership behaviors and team learning.

**Results:**

Results from structural equation modeling analyses suggest that, at a team level, participative leadership behaviors have both a direct association with team learning and are partially mediated by the team’s justice climate.

**Discussion:**

This study contributes to existing literature by offering evidence that the perceptions of justice instilled by leaders play a role mediating participatory leadership and team learning. Moreover, the study supports the idea that leader induced justice perceptions can be considered as an aggregated construct at the team level. From a practical standpoint, the findings imply that team leaders can contribute to create an environment conducive to team learning by treating team members with fairness.

## 1 Introduction

Team learning plays a central role in assisting organizations to adapt to the dynamic and complex business landscapes of today, marked by uncertainty and turbulence (Van Der Vegt and Bunderson, 2005; Edmondson, 2012). Team learning, as defined in this context, refers to a team’s commitment to continuously improve processes and products through systematic knowledge sharing, problem-solving, and collaboration among team members (Edmondson, 2003). Among the critical antecedents of team learning, participative leadership behaviors exhibited by team supervisors stand out (e.g., Sarin and McDermott, 2003; Lundqvist et al., 2023). These behaviors entail leaders integrating team members’ viewpoints into decision-making (Arnold et al., 2000), encouraging employees to exchange and analyze knowledge about their work (Sarin and McDermott, 2003), and promoting collaboration to collectively shape how work is approached (Lorinkova et al., 2013). The link between participative leadership and team learning is considered indirect, with leaders creating conditions that promote team members’ engagement in learning behaviors. Mediators studied in this relationship include psychological safety, team dynamics (e.g., information exchange, collaboration), and trust among team members (Carmeli et al., 2011; Edmondson, 2012; Ye et al., 2019).

Previous literature has also recognized participative forms of leadership as a precursor to team members justice perceptions (e.g., Karam et al., 2019). Justice perceptions refer to the subjective perceptions of fairness that employees have about their workplace, and four key components are often discussed (Colquitt et al., 2013): distributive justice (concerned with outcomes like pay), procedural justice (related to fair decision-making procedures), and interactional justice (comprising interpersonal and informational justice, which involve the quality of treatment and adequacy of explanations for decisions). Leaders’ decisions and actions are often regarded as a primary source of employees’ fairness perceptions at work, as they hold discretionary power in matters like pay, performance evaluations, and promotions (Cropanzano et al., 2007; Scott et al., 2014). It has been suggested that participative forms of leadership influence employees’ justice perceptions by fostering open communication with their subordinates (Larson et al., 1998; Somech, 2005). By enabling employees to voice their concerns and seek explanations regarding their supervisors’ actions and decisions (Wang et al., 2022; Qing and JinHua, 2023), and providing adequate justifications or explanations to employees’ concerns, leaders create the conditions for the emergence of positive justice perceptions among employees (Shaw et al., 2003). Additionally, empirical evidence supports that, when employees perceive they are treated with fairness and respect at work, team learning related dynamics and outcomes emerge (Dayan et al., 2009; Akgün et al., 2010; Gerlach, 2019).

**Figure 1:**
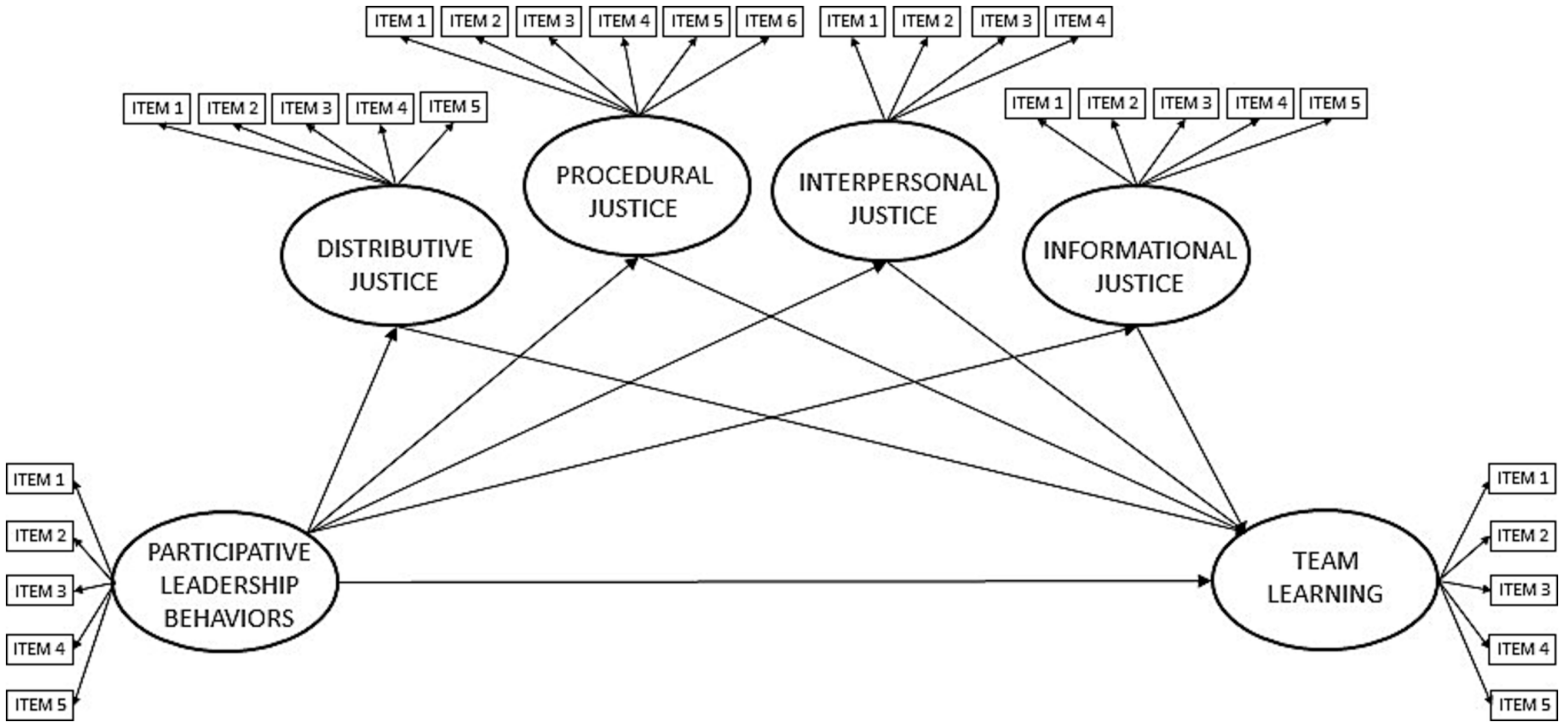
Proposed model 1 predicting direct effects of participative leadership on team learning and the partial mediations of distributive, procedural, interpersonal, and informational justice. Model does not achieve a good fit (CMIN/DF = 2.741, IFI = 0.864, CFI = 0.863, RMSEA = 0.091).

While previous literature suggests that participative leadership enhances employees’ justice perceptions and that these perceptions foster team learning, empirical research on the mediating role of justice perceptions in the relationship between participative leadership and team learning is lacking. Building on the preceding points, and theoretical support from equity theory and social exchange theory, this study’s main objective is to explore how leader-induced fairness perceptions among employees mediate the link between participative leadership and team learning. Figure 1 presents the proposed model, which is discussed in greater detail in subsequent sections.

This study adds to the current body of literature in several ways: first, it addresses a research gap by examining how leaders’ fairness decisions and behaviors serve as a mediation mechanism through which participative leadership influences team learning, thus exploring a previously unexamined mediator. Second, as proposed in the next section, it reaffirms the relevance of equity theory and social exchange theory as theoretical frameworks for understanding how team leaders’ actions influence team learning. Furthermore, it underscores the importance of aggregating leader-induced justice perceptions at the team level. From a practical standpoint, this study highlights the crucial role of fair treatment in promoting team learning. Lastly, this research explores these dynamics within a moderately high-power distance culture like Chile, shedding light on the factors that facilitate team learning in such contexts (e.g., Robert et al., 2000; Parnell, 2010; Pillai et al., 2011; Gupta and Singh, 2015; Anand et al., 2018; Qing and JinHua, 2023).

The next section of this paper introduces the theoretical framework that supports a model proposing a positive relationship between participative leadership behaviors (the independent variable) and team learning (the dependent variable), with partial mediation by team members’ perceptions of justice climate induced by their leader. Following that, the paper details the methodology used to test this model and provides the results. Lastly, the study delves into the theoretical and practical implications, acknowledges its limitations, and draws conclusions.

## 2 Literature review and hypotheses development

### 2.1 Participative leadership behaviors and team learning

To foster team learning, organizations must establish an environment conducive to employees’ engagement in knowledge acquisition and exchange behaviors (Burke et al., 2006). Team leaders are pivotal in fostering team learning by actively encouraging employees’ engagement and participation (Edmondson, 2003; Wagner, 2009; Lundqvist et al., 2023). Indeed, empirical research consistently confirms a positive association between participative forms of leadership and team learning (e.g., Larson et al., 1998; Edmondson, 2003; Huang et al., 2011; Lorinkova et al., 2013).

Prior research has inquired into several mediation mechanisms that elucidate the relationship between participative leadership and team learning. Notably, psychological safety, denoting team members’ belief in their ability to voice opinions without fear of reprisal or humiliation, has emerged as a prominent mediator (i.e., Edmondson, 2012; Ye et al., 2019). Additionally, behavioral integration, encompassing various team dynamics like information exchange, collaboration, and collective decision-making, has been recognized as another substantial mediator in the participative leadership-team learning dynamic (Carmeli et al., 2011; Chiu et al., 2021). Other mediating factors, including trust among team members (i.e., Carmeli et al., 2012), and a market or customer-focused shared mindset among team members (Rustad Bjerke, 2023), have also demonstrated significant mediating roles in the relationship between participatory leadership and team learning. Prior research has placed less emphasis in studying employees’ fairness perceptions of the treatment received by their supervisors as a mediator in the participative leadership-team learning relationship, despite the existence of substantial bodies of research suggesting that team leaders have an influence on employees’ perceptions of fairness (Karam et al., 2019), and that employees’ perceptions of fairness facilitate team learning (e.g., Dayan and Colak, 2008; Akgün et al., 2010; Gupta and Singh, 2015).

The theoretical rationale and prior empirical findings from the literature to substantiate the proposed mediation pathways will be presented next (see Figure 1). First, the connections that clarify how the independent variable (participative leadership) influences the mediators (leader-induced justice dimension perceptions) will be examined. Later, the relationships between these mediators and the dependent variable (team learning) will be explored.

### 2.2 Participative leadership as an antecedent of leader induced justice perceptions.

Fairness theory provides a suitable theoretical framework for understanding how participative leadership behaviors relate to justice perceptions. According to this theory, individuals’ reactions to their superiors’ behaviors and decisions are influenced by the explanations they construct, which can be shaped by explanations provided by others (Folger and Cropanzano, 2001). These reactions are described in terms of blame or accountability, and they involve comparing what happened with what could and should have happened (Folger and Cropanzano, 2001). If individuals perceive that there were alternative courses of action available to the decision-maker, they may attribute unfairness and blame. Specifically, participative leaders are characterized for fostering open communication environments with their subordinates, encouraging employees to voice their concerns and seek explanations for work-related issues (Larson et al., 1998; Somech, 2005). By enabling employees to express their concerns, and by providing explanations and answers to their inquiries, supervisors can offer arguments that justify or explain their actions decisions that have an impact on their subordinates (Shaw et al., 2003). Indeed, Colquitt and Rodell (2015) coined the concept of ‘supervisor-focused fairness,’ in reference to employees’ justice perceptions originated from their supervisors’ actions and decisions. Furthermore, because of the role played by direct supervisors in facilitating fairness judgments among their employees, it can be expected that justice perceptions should vary across different teams and from one leader to another. Finally, in a meta-analysis study, Colquitt et al. (2013) found that leader induced justice measures, when compared to organizational justice measures, revealed stronger effect sizes over outcome measures, arguing that supervisor behaviors may be more directly observable and judged than company policies and actions. Next, the use of fairness theory to explain the participative leadership behaviors - justice perceptions link, is extended to each specific dimension of employees’ justice perceptions.

#### 2.2.1 Link between participative leadership and employees’ perceptions of distributive justice

Research emphasizes the critical role of supervisors in shaping employees’ perceptions of distributive justice, as they often have discretion in determining pay (Bailey et al., 2011). Fairness theory proposes that supervisors must offer clear explanations regarding the connection between their efforts and the outcomes they can expect from their work (Hartmann and Slapničar, 2012). Participative leadership, with its open communication setting, should enhance perceptions of distributive justice, by enabling employees to express their concerns and receive explanations regarding the link between their efforts and outcomes. Empirical evidence consistently demonstrates a positive association between participative leadership and favorable distributive justice perceptions among subordinates. For instance, in a study involving Chinese workers, Wang et al. (2011) discovered that leaders who actively encourage effective communication with subordinates are linked to more positive distributive justice perceptions. Moreover, Pillai et al. (1999) found that, for employees to perceive fairness, supervisors must instill in them the belief that the outcomes they receive for their work are appropriate. Hence, it is proposed that participative leadership behaviors will foster favorable perceptions of distributive justice among employees.

#### 2.2.2 Participative leadership and procedural justice perceptions

Managers play a pivotal role in performance evaluations and promotions (Scott et al., 2014). Fairness theory suggests that supervisors open communication, addressing employee concerns about work procedures and decisions, can promote positive perceptions of procedural justice among employees, idea that is supported by empirical evidence. For instance, in a study involving strategic decision-making teams, Korsgaard et al. (1995) found that leaders’ consideration of team members’ opinions positively correlates with members’ perceptions of procedural fairness. Also, Avery and Quiñones (2002) show that individuals who perceive they have a voice opportunity experience a significant increase in procedural justice perceptions. Similarly, in a quasi-experimental study involving a large sample of Finnish public sector employees, Linna et al. (2011) found that organizational interventions promoting employee participation led to an increase in employees’ perceptions of procedural justice. Therefore, it is proposed that participative leadership behaviors will promote positive procedural justice perceptions among employees.

#### 2.2.3 Participative leadership and interactional justice perceptions

In line with fairness theory, providing employees opportunities to participate should boost their sense of respect from leaders (Ghaffari et al., 2017), feelings of importance within the organization (Jago, 2017), and the ability to voice their opinions, thereby promoting interpersonal justice. Additionally, this mutual engagement allows employees access to more information about decisions that affect them, enhancing their perception of informational justice. Empirical evidence confirms a positive relationship between participative leadership and interactional justice perceptions. For example, Gavin et al. (1995) found that employees’ perceptions of interactional justice tend to increase when their supervisors adopt a cooperative problem-solving approach. Likewise, Cho and Dansereau (2010) discovered that leaders who promote empowering behaviors among their followers tend to generate perceptions of interactional justice among them. Finally, Ghaffari et al. (2017) identified a positive association between participative leadership and employees’ sense of being respected by their leaders. Therefore, it is suggested that participative leadership behaviors foster positive perceptions of interactional justice among employees.

Finally, while previous literature has primarily examined justice perceptions at individual or organizational levels, recent research highlights the influence of team members’ opinions and responses on each other’s justice perceptions. As such, some authors advocate the concept of ‘justice climate,’ referring to the aggregate justice perceptions of a group (Naumann and Bennett, 2000). In consequence, this study assesses for the convenience of aggregating ‘leader induced’ justice perceptions at a team level (Colquitt and Rodell, 2015).

### 2.3 Justice perceptions relation to team learning

As illustrated in Figure 1, justice perceptions (the mediators) must also influence team learning (the outcome variable) to mediate the participative leadership-team learning relationship. These connections can be elucidated by social exchange theory, which posits that employees regard their relationships with employers as social exchanges grounded in reciprocity (Cropanzano and Mitchell, 2005). if employees feel fairly treated, they will consider themselves in a reciprocal employment relationship and retribute by engaging in cooperative voluntary behaviors. Lind and Tyler (1988) argue that justice perceptions serve as precursors of voluntary cooperative behaviors by signaling to employees that their self-interests are protected, and that they are valued and respected by their managers. Furthermore, prior research demonstrates that fairness in the treatment of employees plays a significant role in encouraging voluntary cooperative behaviors, including organizational citizenship behavior (Colquitt et al., 2013), team members assisting one another (Chattopadhyay, 1999), and readiness to change (Metwally et al., 2019).

Considering that team learning can also be viewed as a cooperative, voluntary behavior—exemplified by knowledge sharing, contributing ideas, or helping others learn on the job (Van den Bossche et al., 2006)—it can be argued that team learning may be utilized as a form of retribution by employees when they perceive fair treatment from their supervisors. Even when such behavior is not directly compensated, employees may regard team learning as an appropriate response to positive justice perceptions. This argument can be extended to the relationship of each specific justice perceptions dimension with team learning.

#### 2.3.1 Link between distributive justice and team learning

By extending social exchange reciprocity principle, it can be argued that when team members perceive receiving a fair pay, they will be more inclined to engage in team learning. Empirical evidence is consistent with this argument. For example, in a sample of business units from companies in the United States and Great Britain, Cowherd and Levine (1992) find evidence of a positive relationship between distributive justice perceptions and cooperative behaviors that enhance product quality, such as offering help and correcting errors. Dayan et al. (2009) also find that distributive justice is a significant antecedent of team learning in a sample of new product development projects in Turkey. As well, Lin (2007) shows that distributive justice serves as an antecedent of tacit knowledge sharing in a sample of Taiwanese employees. Hence, it is proposed that the perceptions of distributive justice will foster team learning.

#### 2.3.2 Procedural justice relation to team learning

Consistent with the reciprocity principle of social exchange theory, numerous authors contend that perceptions of procedural justice significantly influence employees’ readiness to engage in collaboration, knowledge-sharing, innovation in product development, and the display of proactive, risk-taking behaviors (Li et al., 2007; Dayan and Colak, 2008; De Clercq et al., 2010; Fang and Chiu, 2010; Gupta and Singh, 2015; Akram et al., 2017). Specifically, in a sample of new product development teams in Turkey, Akgün et al. (2010) show that procedural justice perceptions serve as an antecedent of team learning. Furthermore, Boudrias et al. (2010) show that, the relation between a managers’ participative decision-making style, and his/her subordinates work improvement and collaboration efforts, is stronger when high levels of procedural justice are present. Therefore, it is suggested that perceptions of procedural justice will promote team learning.

#### 2.3.3 Interactional justice relation to team learning

In an environment where employees feel well-informed, and treated with respect and dignity by their supervisors, the reciprocity principle of social exchange theory predicts that they will be more willing to engage in voluntary behaviors such as team learning. This connection is supported by previous empirical evidence. For instance, Cho and Dansereau (2010) provide evidence that interpersonal justice acts as a mediator in the relationship between a leader’s individualized consideration and their subordinates’ organizational citizenship behavior. Likewise, research has established a link between perceptions of interactional justice and team members’ creative behaviors (e.g., George and Zhou, 2001; Khazanchi and Masterson, 2011), and with employees’ motivation for learning and knowledge sharing (Gerlach, 2019). Consequently, it is expected that perceptions of interactional justice will facilitate team learning.

### 2.4 Hypotheses

The preceding sections have presented both a theoretical framework and empirical evidence supporting each link in the mediation model depicted in Figure 1. This model proposes that participative leadership influences various dimensions of employees’ justice perceptions, which, in turn, impact on team learning. However, because the influence of direct leadership on outcome measures may also be carried through other mediators, such as psychological safety, and trust among team members (i.e., Carmeli et al., 2012; Edmondson, 2012), a partial rather than a complete mediation of justice is expected. The hypotheses proposed in Model 1 can be summarized as follows:

*H1*: Participative leadership behaviors are positively related to team learning.

*H2*: Distributive justice climate mediates the relation between participative leadership behaviors and team learning.

*H3*: Procedural justice climate mediates the relation between participative leadership behaviors and team learning.

*H4*: Interpersonal justice climate mediates the relation between participative leadership behaviors and team learning.

*H5*: Informational justice climate mediates the relation between participative leadership behaviors and team learning.

## 3 Methods

### 3.1 Sample

Data was collected from 211 teams, from different Chilean organizations, using a convenience sample. Even though convenience sampling is limited by issues of representativeness, personal contacts help ensure higher response rates. Using Hollenbeck et al. (2012) three dimensional scaling model for team description, the surveyed teams can be categorized as follows: (a) All teams exhibited authority differentiation, as it was a requirement for their participation to have a formal team manager or leader; (b) Teams also had a high degree of temporal stability, as both leaders and team members were required to have held their respective roles for a minimum of one year; (c) Teams were also required to be part of a specific functional area within their organizations, have work interdependence among team members, and work toward pursuing team goals or objectives assigned by the organization. These later requisites constrained skills differentiation among team members. The functional specialization to which teams were affiliated were distributed as following: finance and accounting 15.6%, marketing 6.2%, sales and commerce 7.2%, human resources 5.2%, research and development 6.6%, production 17.1%, procurement 4.7%, quality assurance 2.8%, customer service 7.6%, operations management 7.6%, logistics 8.1%, technology and equipment 8.5%, and strategy and planning 2.8%. Team size ranged from four to thirteen individuals, with an average of 8,29 team members, within the higher end size of what has been traditionally conceptualized as small teams in literature (Hollenbeck et al., 2012). Team gender diversity, measured as the percentage of female team members, averaged 31,2% (range from 0 to 100%). Four teams were composed of only females, 16 of only males, and 191 of mixed gender. Basically, the teams in the sample closely align with Cohen and Bailey’s (1997) description of ‘work teams’, characterized as stable units directed by a supervisor, primarily focused on providing internal or external services or producing goods. These types of teams are distinct from other forms of teams, such as cross-functional and temporal project teams (Cohen and Bailey, 1997; Hollenbeck et al., 2012). The teams worked within companies at a diverse range of industries, including retail trade (11%), finance (9%), tourism (8%), professional services (10%), agriculture (9%), energy (6%), telecommunications (8%), transportation (9%), manufacturing (10%), technology (7%), construction (8%), and mining (6%).

A total of 807 responses for the leaders’ participative behaviors and justice climate measures were collected, with an average of 3.82 responses per team, that is 46% of team members responses per team (range: 33–57%). This sub-sample averaged 35.78 years old, and 29.4% of them were female. For the team learning measure 856 responses were collected, with an average of 3.97 responses per team, that is 48% of team members responding per team (range: 33–67%). This sub-sample averaged 33.9 years old, and 31.6% of them were female. The highest attained educational level reported by participants was in a 6.6% high school, 30.4% technical degrees, and 63.0% professional degrees.

### 3.2 Procedure

The initial contact with teams was secured through managers in executive education programs at a well-known business school in Chile. Contacts were requested to provide links for all team members. Respondents were instructed to evaluate their supervisors and teams for developmental purposes. Surveys were distributed in a paper format and delivered to respondents in an envelope. Responders then returned their completed surveys in sealed envelopes to the researcher. Participation was voluntary, and subjects received no monetary compensation or otherwise. Participants were assured and secured confidentiality and anonymity. Several measures were taken to decrease the potential threat of common method variance and multi-collinearity problems (Podsakoff et al., 2003; Beckstead, 2012). First as described, a split-half sampling approach was employed, where approximately half of team members responded the participative leadership and justice climate measures, and the other half responded the team learning measure, method that has been utilized in similar research contexts (Ostroff et al., 2002; Gerhart, 2008; Kehoe and Wright, 2013). Secondly, distinct response range formats were applied to the Likert scales used for the participative leadership and justice climate measures. Finally, to assess any potential common method variance among respondents of the participative leadership behaviors and justice scales, the marker-variable technique proposed by Lindell and Whitney (2001) was employed.

### 3.3 Measures

Perceptions of distributive justice were assessed using the Distributive Justice Index by Price and Mueller (1986), a five-item scale measuring the fairness of rewards in relation to performance, responsibilities, education, and effort. Sample items include ‘My supervisor rewards me fairly considering my responsibilities’ and ‘My supervisor rewards me fairly for the effort I put into my work.’ Participants rated these items on a Likert scale ranging from 1 (strongly disagree) to 7 (strongly agree). Procedural and interactional justice were measured using scales developed by Niehoff and Moorman (1993). Six items evaluated the fairness of procedural decisions made by supervisors, focusing on information gathering, employee voice, and appeals processes. Sample items include ‘My supervisor ensures that all employee concerns are heard before decisions are made’ and ‘Job decisions by my supervisor are unbiased.’ Nine items assessed the degree to which employees were treated with dignity and respect and received clear explanations for workplace decisions. Sample items include ‘My supervisor treats me with dignity and respect’ and ‘My supervisor provides clear explanations for decisions affecting me at work.’ Participants used the same Likert scale mentioned earlier. To gauge participative leadership behaviors, five items from Arnold et al.’s (2000) participative leadership behaviors scale were adapted. Sample items include ‘Our supervisor encourages work group members to express ideas/suggestions’ and ‘Our supervisor promotes information exchange among work group members.’ Participants rated these items on a Likert scale ranging from 0 (absolutely disagree) to 10 (completely agree). Team learning was measured using a validated scale by Edmondson (1999), consisting of five items rated on a Likert scale ranging from 1 (never) to 5 (always). Sample items include ‘This team dedicates time to finding ways to enhance its performance’ and ‘When necessary, this team seeks new information to improve our work.’ Additionally, a control variable indicating the leader’s gender (0 for female and 1 for male) was included in the analysis due to its known impact on psychological and organizational outcomes (Paris et al., 2009). To evaluate common method variance, the marker variable method proposed by Lindell and Whitney (2001) was employed. This method involved three survey items adapted from Shih (2004) to measure attitudes toward using the Internet, which served as a conceptually unrelated marker variable. Example items include ‘I like to use the Internet’ and ‘It is a pleasure for me to use the Internet.

To ensure items accuracy and clarity the translation of survey items followed Harkness (2003) methodology, where a separate translator, reviewers, and recipients were involved. To validate the translated version, a pilot study was conducted with a sample of respondents. They assessed the readability and comprehension of the translated items. When needed, item corrections were made based on their feedback.

## 4 Results

Table 1 shows basic statistics and correlations among the variables at an individual level, before team level aggregation. Participative leadership behavior presents a positive significant relation with team learning and the justice dimensions. Additionally, the justice indices also display positive and significant correlations with team learning and with each other. Noteworthy, there is a small positive correlation (0.17, *p* < 0.05) between the presence of female leaders and team members’ positive perceptions of distributive justice. Table 1 displays the Cronbach’s alpha reliability scores for all scales, demonstrating internal consistency with values above the recommended 0.70 threshold (Nunnally, 1978). Moreover, the item values exhibit acceptable asymmetry (ranging from min = −1.5 to max = 0.1) and kurtosis (ranging from min = −0.8 to max = 2.0), indicative of a normal univariate distribution (George and Mallery, 2010).

**Table 1:** Means, standard deviations and correlations for all variables.

	*M*	*SD*	1	2	3	4	5	6	7	8	9
1. Leader participative behaviors	6.85	1.24	(0.93)								
2. Distributive justice	4.92	0.86	0.54	(0.93)							
3. Procedural justice	5.39	0.71	0.58	0.66	(0.87)						
4. Interpersonal justice	5.65	0.72	0.49	0.55	0.69	(0.89)					
5. Informational justice	5.79	0.65	0.55	0.68	0.79	0.82	(0.92)				
6. Team learning	3.37	0.57	0.48	0.47	0.48	0.35	0.41	(0.87)			
7. Leader gender	0.75	0.43	−0.10	−0.17	−0.06	−0.09	−0.05	−0.11			
8. Team gender diversity	0.31	0.21	−0.01	−0.08	−0.10	−0.01	−0.01	−0.02	−0.09		
9. Team age diversity (years)	9.04	0.21	0.08	0.06	0.04	0.07	0.12	0.02	0.07	−0.07	
10. Team size	8.29	2.07	0.03	−0.07	−0.11	−0.09	−0.07	−0.02	−0.9	0.04	−0.08

Cronbach’s alpha for all scales is presented on the diagonal in parenthesis.

Gender, 1 = male, 0 = female.

Correlations > |0.13| are significant at *p* < 0.05; correlations > |0.18| are significant at *p* < 0.01.

To assess common method variance using the marker variable method (Lindell and Whitney, 2001), the items measuring attitudes toward using the Internet (Shih, 2004) were averaged to create an ‘attitudes toward using the Internet’ index (Cronbach’s Alpha = 0.78). Correlations between this index and the research variables ranged from 0.020 to 0.040, all of which were non-significant. Following Lindell and Whitney (2001) procedure, the second smallest correlation (r = 0.028) between the marker variable and the research variables was used to control for potential common method variance. Subsequently, all correlations among research variables remained significant at *p* < 0.001, indicating that common method variance did not substantially affect results.

The present study tests for Naumann and Bennett’s (2000) argument that individual justice perceptions when analyzed at a team level should be aggregated and referred to as justice climate. Similar rationale has been applied to the aggregation of team learning (Van Der Vegt and Bunderson, 2005; Chiu et al., 2021) and leadership measures (Lord et al., 2001). To justify data aggregation at the team level, Rwg (j), ICC (1), and ICC (2) statistics were calculated (see Table 2). Rwg (j) values for all variables exceeded the accepted threshold of 0.70, supporting data aggregation (Biemann et al., 2012). ICC (1) indicates the proportion of total variance explained by group membership, and our values exceeded the median of 0.12 reported by James (1982). Furthermore, ICC (2) scores fall within the upper range of moderate agreement, as suggested by Biemann et al. (2012). Therefore, responses from all team members were averaged to create team-level scores.

**Table 2:** Unit-level aggregation statistics for all measures.

	Rwg(j)	ICC(1)	ICC(2)
Leaders participative behaviors	0.85 (*F* = 2.95)	0.34	0.66
Distributive justice	0.71 (*F* = 1.56)	0.13	0.36
Procedural justice	0.80 (*F* = 1.91)	0.19	0.41
Interpersonal justice	0.87 (*F* = 1.81)	0.18	0.45
Informational justice	0.82 (*F* = 1.89)	0.19	0.47
Team learning	0.82 (*F* = 3.08)	0.34	0.68

All *F* values are significant at *p* < 0.01.

A two-stage analysis was conducted using AMOS 28. First, a confirmatory factor analysis (CFA) assessed the discriminant validity of the independent and mediator variables measures. Subsequently, structural equation modeling (SEM) was employed to evaluate competing structural models (Kline, 2011). Due to the documented high inter-correlations between justice dimensions in prior research (Colquitt et al., 2013), the fit of five different factor structures was tested. These included a one-factor model, a two-factor model with participative leadership and justice items, a three-factor model separating distributive justice from procedural and interpersonal justice, a four-factor model with distinct distributive, procedural, and interpersonal justice factors, and a five-factor model distinguishing between the interpersonal and informational subcomponents of interactional justice.

Table 3 displays the results for all tested CFA models. Among these models, the five-factor model demonstrated the best fit to the data (CMIN/DF = 2.075, CFI = 0.936, IFI = 0.935, RMSEA = 0.072, Confidence Intervals: Low 90 = 0.063 & High 90 = 0.080), outperforming models with fewer factors. While some modification indices could potentially improve model fit, they were not considered, as they lacked a theoretical basis and likely reflected idiosyncratic sample characteristics or measurement error (MacCallum et al., 1996). All items in the best-fitting model exhibited significant loadings on their respective factors, with factor loadings ranging from 0.62 to 0.93 and an average loading of 0.82.

**Table 3:** Confirmatory factor analysis for several factor structures for independent and mediation variables items.

	*χ* ^2^	df	CMIN/DF	CFI	IFI	RMSEA	LO 90 / HI 90
Model 1 (1-factor)	1655.91	275	6.021	0.687	0.685	0.155	0.147 / 0.162
Model 2 (2-factors)	1202.09	274	4.387	0.790	0.788	0.127	0.120 / 0.134
Model 3 (3-factors)	761.30	272	2.799	0.889	0.888	0.093	0.085 / 0.100
Model 4 (4-factors)	639.38	269	2.377	0.916	0.915	0.081	0.073 / 0.089
Model 5 (5-factors)	549.85	265	2.075	0.936	0.935	0.072	0.063 / 0.080

All *χ*^2^ values significant at *p* < 0.01.

Model 1 (see Figure 1), which assesses the parallel mediation of each justice dimension using the five factors confirmed in the CFA, yielded unsatisfactory fit indices (CMIN/DF = 2.741, IFI = 0.864, CFI = 0.863, RMSEA = 0.091, Confidence Intervals: Low 90 = 0.085 & High 90 = 0.098). when analyzing these indices with Hu and Bentler (1999) criteria. Due to this inadequate fit, alternative model were explored to better explain the data.

In separate analyses (not shown here), the individual mediation path for each justice dimension was tested, while excluding the other paths from the model. In these analyses, all paths were statistically significant. It’s worth noting that previous meta-analytic research on justice perceptions has highlighted the high correlation among these dimensions (Colquitt et al., 2001, 2013). This can lead to potential issues of multicollinearity in structural models. Colquitt and Rodell (2015) propose that these high correlations may stem from shared variance, reflecting a general sense of justice attributed by employees to the perceived source of justice (e.g., their team leader), as well as specific variance unique to each justice dimension. To address this issue, two additional models based on Colquitt and Rodell (2015) are explored, aiming to account for the intercorrelations among justice dimensions and potentially mitigate the overshadowing of mediation effects seen in Model 1.

**Figure 2:**
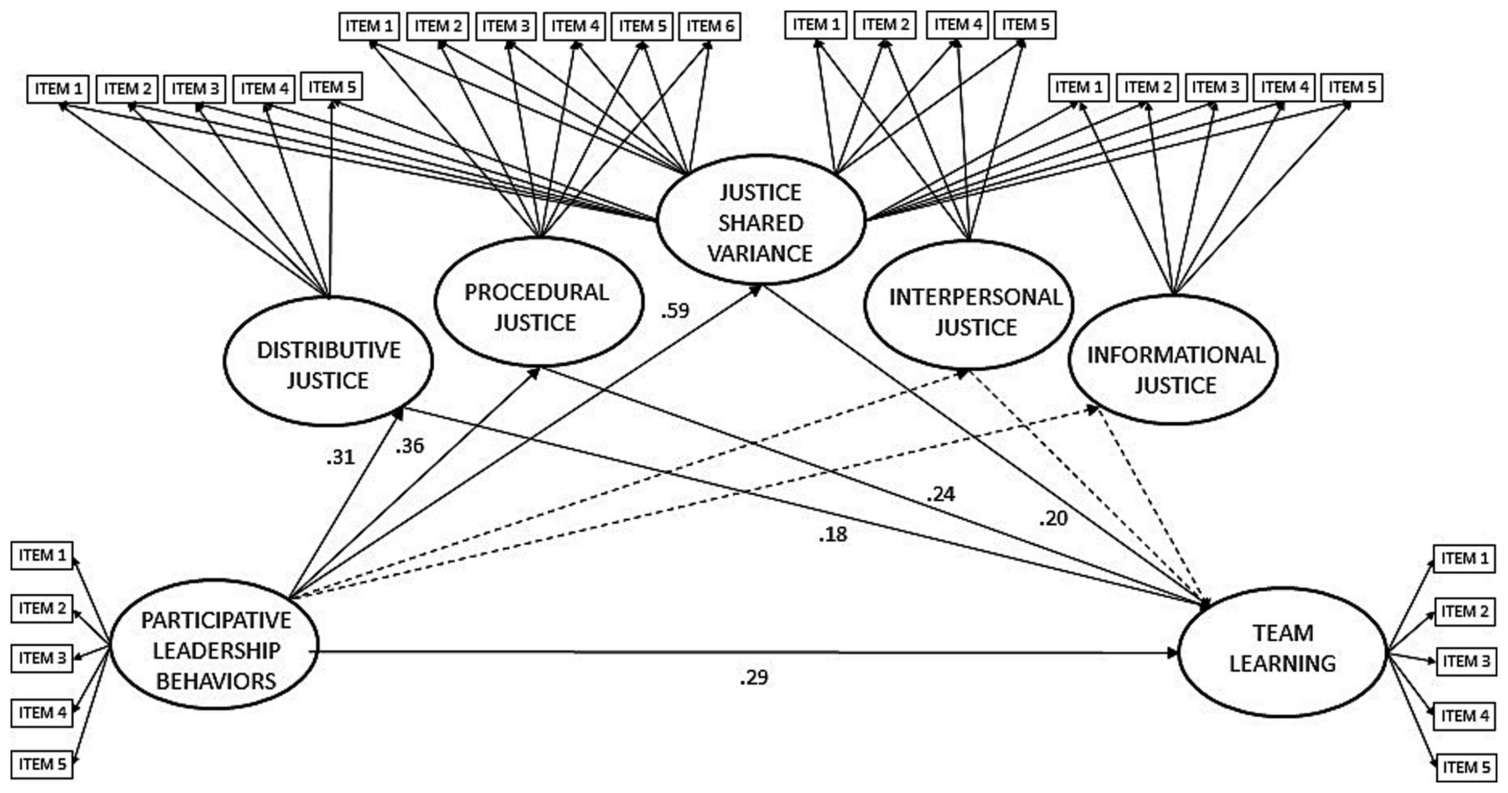
Standardized coefficients for model 2 predicting direct effects of participative leadership on team learning and the partial mediations of distributive, procedural, interpersonal, and informational justice, as well as a general justice shared variance. (CMIN/DF = 1.887, IFI = 0.935, CFI = 0.934, RMSEA = 0.065). Segmented lines represent non-significant paths.

Model 2 (refer to Figure 2) is an adaptation of Model 1, incorporating a “shared justice variance” factor across all justice items alongside each justice dimension’s specific variance. This model acknowledges the presence of partially shared variance among justice dimensions, representing a general justice component, while preserving dimension-specific variance. Model 2 demonstrates a good fit for the data (CMIN/DF = 1.887, IFI = 0.935, CFI = 0.934, RMSEA = 0.065, Confidence Intervals: Low 90 = 0.058 & High 90 = 0.072). Structural equation analysis of Model 2 reveals several significant relationships. Participative leadership behaviors are positively associated with team learning (*β* = 0.29, *p* < 0.01), distributive justice (*β* = 0.31, *p* < 0.01), procedural justice (*β* = 0.36, *p* = 0.01), and “general perceptions of justice” (*β* = 0.59, *p* < 0.01). However, they are not related to informational justice (*β* = 0.10, *p* = 0.75) and interpersonal justice (*β* = 0.02, *p* = 0.87). Additionally, distributive justice (*β* = 0.18, *p* < 0.01), procedural justice (*β* = 0.24, *p* < 0.02), and the “general perceptions of justice” dimension (*β* = 0.20, *p* < 0.02) show positive relationships with team learning, while interpersonal justice (*β* = 0.07, *p* = 0.34) and informational justice (*β* = −0.03, *p* = 0.34) are not related to team learning. Therefore, the results of Model 2 support Hypotheses 1, 2, and 3, indicating a direct effect of participative leadership behaviors on team learning and mediations through distributive and procedural justice. Furthermore, Model 2 introduces an additional mediation path through “general perceptions of justice.

**Figure 3:**
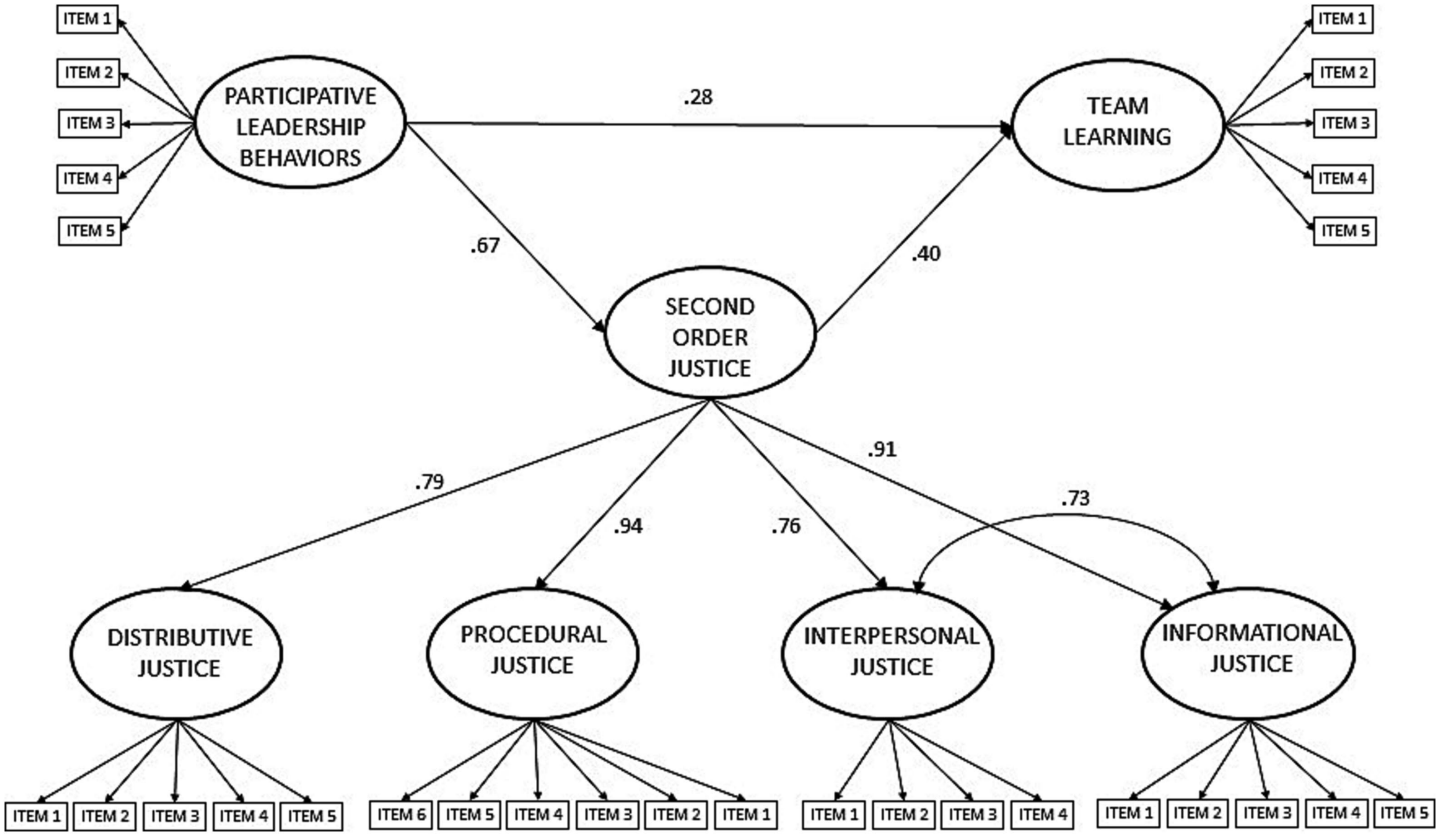
Standardized coefficients for model 3 predicting direct effects of participative leadership on team learning and the partial mediations of a second order factor grouping all justice dimensions (CMIN/DF = 1.957, IFI = 0.925, CFI = 0.924, RMSEA = 0.066).

Model 3 also aims to address the high correlations among justice dimensions. This model introduces a second-order factor fed by the four justice facet dimensions, creating a single mediation path through which participative leadership is mediated by justice (refer to Figure 3). Model 3 allows for the covariance of error terms between interpersonal and informational justice dimensions, as suggested by a modification index. This covariance is theoretically relevant since these two justice dimensions are highly related conceptually and statistically, sometimes forming a single interactional latent factor (Colquitt and Rodell, 2015). Model 3 demonstrates a good fit for the data (CMIN/DF = 1.957, IFI = 0.925, CFI = 0.924, RMSEA = 0.066, Confidence Intervals: Low 90 = 0.060 & High 90 = 0.075). Structural equation results for Model 3 indicate that the four justice facet dimensions load significantly on the second-order justice factor (distributive: *β* = 0.79, *p* < 0.01, procedural: *β* = 0.94, *p* < 0.01, interpersonal: *β* = 0.76, *p* < 0.01, and informational: *β* = 0.91, *p* < 0.01). Furthermore, participative leader behaviors are positively related to team learning (*β* = 0.28, *p* < 0.01) and the second-order justice factor (*β* = 0.02, *p* < 0.67). Both participative leader behaviors (*β* = 0.28, *p* < 0.01) and the second-order justice factor (*β* = 0.02, *p* < 0.40) are positively related to team learning. Consequently, Model 3 provides support for the direct effect of participative leadership behaviors on team learning, as well as the presence of a second-order justice factor. In summary, Models 2 and 3, which account for significant correlations among justice dimensions, exhibit better overall fit to the data.

Boudrias et al. (2010) proposed the possibility of a moderation effect of organizational justice on the relationship between participative leader behaviors and team learning. Post-hoc analyses were conducted to explore this potential moderation effect for each dimension of justice climate. However, none of the justice dimensions—distributive, procedural, interpersonal, and informational justice—showed significant moderation effects. The ΔR2 values ranged from 0.000 to 0.006, and the corresponding *p*-values were all greater than 0.168, 0.798, 0.446, and 0.180, respectively. One possible explanation for the significance of the mediation model and the lack of significance in the moderation model could be related to the nature of the justice measures used in this study. The measures used in the present study assessed leaders’ induced justice perceptions, suggesting that employees might perceive justice perceptions because of supervisors’ behaviors and decisions. In contrast, in studies where justice measures are framed as emanating from organizational policies and practices, as is more common in previous research (van Knippenberg et al., 2007), justice perceptions are not necessarily linked by employees to managers’ behavior but rather to organizational policies and procedures.

Finally, to assess the impact of control variables on the SEM models, teams’ industry (e.g., mining, professional services, etc.) and functional specialization categories (Human Resources, Marketing, etc.) were converted into dummy variables. Here, ‘1’ denoted the team’s affiliation with a specific category, while ‘0’ signified no affiliation. Subsequently, correlations were calculated between the main study variables and all control variables, including leader’s gender, teams’ gender diversity, age diversity, team size, and the industry and functional specialization dummies. In most cases these correlations were not significant. However, there were a few exceptions: a significant correlation was found between female leaders and distributive justice (R2 = 0.17, *p* < 0.05), indicating that female leaders were perceived as exhibiting more distributive justice; a significant correlation was observed between the ‘professional services’ firms and distributive justice of (R2 = –0.17, *p* < 0.05), suggesting that ‘professional services’ firms were perceived as having less distributive justice; a significant correlation of R2 = 0.15 (*p* < 0.05) was detected between the ‘telecommunication’ firms dummy variable and team learning, indicating that ‘telecommunication’ firms were perceived as having more team learning; construction firms (R2 = 0.15, *p* < 0.05) and sales teams (R2 = 0.15, *p* < 0.05) dummies exhibited a significant correlation with age diversity; and finally, a significant correlation was found between sales teams and the presence of female leaders (R2 = 0.14, *p* < 0.05). When the control variables and industry and teams’ function dummies with a significant relationship with a study variable were incorporated into Models 1, 2, and 3, the results of the SEM analyses remained consistent.

## 5 Discussion

### 5.1 Theoretical and conceptual contributions

This study contributes to team learning literature by including team members’ leader-induced justice perceptions as a mediation path in the participative leadership – team learning relation discussion. Previous literature has identified several mediation mechanisms that help explaining the participative leadership – team learning relation, such as team dynamics, trust, and psychological safety. This study results provide strong support for fairness theory as a suitable theoretical framework to explain how participative leadership behaviors contribute to the development of justice perceptions among team members. The argument here presented is that the enhancement of the communication process with their team leader, that is facilitated through participation, helps employees to better understand the fairness of rewards allocated to them, the procedural decisions with which their team leaders affect them, and the way they are treated, regardless of the outcome. The findings of this study also lend support to the reciprocity principle within social exchange theory as a theoretical framework to explain the association between team members’ justice perceptions and team learning. According to this principle, when team members perceive fair treatment within their teams, they are more inclined to reciprocate by exchanging ideas and providing support to their fellow team members. Essentially, the sense of fairness experienced by team members motivates them to engage in cooperative behaviors that contribute to team learning. These results align with findings in other research areas, demonstrating that justice perceptions can act as antecedents to various forms of voluntary behavior by employees, including organizational citizenship behavior (Colquitt et al., 2013; Moorman and Byrne, 2013). In essence, the study highlights the role of justice perceptions as a driving force behind cooperative and voluntary behaviors within teams, ultimately promoting team learning.

Furthermore, the findings of this study underscore the relevance of leadership styles that prioritize ethical behavior, such as ethical leadership and equity leadership (Den Hartog, 2015; Metwally et al., 2019; Kim et al., 2023). These leadership approaches emphasize that leaders’ ethical conduct and decisions play a vital role in creating a conducive environment for employee well-being and the effectiveness of teamwork. Overall, this research reinforces the idea that ethical leadership is not only morally important but also positively influences team outcomes like team learning. Furthermore, this study has several similarities with equity leadership in education. Equity leaders are characterized by openness and shared decision-making, challenging unjust and discriminatory practices, and assessing inequities among those leaded (Kim et al., 2023). These characteristics are similar to those facilitated by participative leadership behaviors which allow team members to inquire for explanations about how they are treated by their supervisors. Like results in this study, findings in equity leadership suggest that leaders’ equity-centered mindset and practices facilitate learning among their followers.

This study also contributes to the literature on justice perceptions and their modeling in research. First, it provides empirical support for the four-dimensional structure of justice perceptions proposed by Colquitt et al. (2001), encompassing distributive, procedural, interpersonal, and informational justice. This finding reinforces the validity of this widely accepted framework for understanding justice perceptions. Second, the study lends support to models that acknowledge the high intercorrelations among justice dimensions, which in this study were disaggregated into a general sense of justice component associated with the perceived source of justice (in this case, team leaders) and dimension-specific variances. Model 2, which allows justice items to load on both specific dimensions and a shared justice factor, demonstrates a better fit to the data. This aligns with the conceptual framework proposed by Colquitt and Rodell (2015), suggesting that justice perceptions encompass both specific and shared variance components. Third, the study contributes to the conceptualization of justice perceptions by demonstrating the relevance of considering them at an aggregated team level. This approach acknowledges that team members influence each other’s perceptions of fairness in the workplace and underscores the importance of leader-induced justice perceptions at the team level. The interpersonal nature of the leader-team member relationship highlights the significance of examining justice perceptions collectively within teams.

The discussion of the cultural context and its potential impact on leadership styles and behaviors is necessary when interpreting the results of this study. The findings that participative leadership behaviors can be effective in a moderately high power distance culture like Chile are interesting and contribute to the understanding of leadership across diverse cultural settings. It could be expected that employees in high-power distance cultures may be less inclined to express their opinions or engage in participative processes, because of the hierarchical structures where they work (Hofstede and Hofstede, 1991). However, this study aligns with previous research that, even in high power distance cultures, there can be a preference for more participatory leadership when given the opportunity (Robert et al., 2000), and that employees’ justice perceptions at work can be facilitated by participative forms of leadership, as has been noticed in several studies previously conducted in high power distance cultures (Somech, 2006; Parnell, 2010; Gupta and Singh, 2015; Qing and JinHua, 2023). Thus, these studies suggest that leadership styles are not solely determined by cultural norms but can also be influenced by how leaders treat their team members. When leaders exhibit participative behaviors, it encourages employees to engage more actively and contribute to team learning, even when such behaviors might initially appear as countercultural.

### 5.2 Limitations

This study, while providing valuable insights, does have certain limitations that should be taken into consideration when interpreting its findings. First, the sample for this study was obtained based on the availability of contacts within companies, which may limit the generalizability of the results. The use of convenience sampling may introduce biases, and the findings may not be representative of all organizations. Second, as previously stated, the study was conducted in Chile, which has its own unique cultural characteristics, such as a high-power distance dimension. This cultural context may influence the way participative leadership is perceived and practiced. Consequently, the generalizability of the findings to other cultural contexts should be approached with caution. Third, the study utilized a cross-sectional design, which allows for the examination of relationships between variables at a single point in time but does not permit causal inferences. Fourth, while the mediation models proposed in the study provide valuable insights, it’s important to acknowledge that mediation does not imply causality. Other unmeasured variables or alternative causal pathways may influence the relationships between participative leadership, justice perceptions, and team learning. Fifth, the study focused on specific variables, such as participative leadership, justice perceptions, and team learning. While these are important factors, other variables not considered in the study may also play a role in the mediation under study. Finally, the study explored potential moderation effects of justice dimensions but did not find significant results. However, moderation effects can be context-specific, and further investigation might be needed to fully understand their role. Future research can build upon this work by addressing these limitations and exploring these relationships in diverse cultural and organizational contexts.

### 5.3 Managerial implications

This study offers several practical implications for team leaders and organizations looking to enhance team learning and foster a fair and just work environment. Team leaders can play a pivotal role in promoting team learning by encouraging participation among team members. Training team leaders in the use of practices that facilitate participation among team members should help members engage in team learning. Furthermore, team leaders should receive training in organizational justice principles (Greenberg, 2011). This training can help them understand the importance of fairness in decision-making processes and how their actions and decisions impact team members’ perceptions of justice. Training should emphasize the use of consistent and transparent rules, involving team members in key procedural decisions, and providing clear explanations for decisions affecting them. This can contribute to the development of a positive justice climate within the team. Finally, by recognizing the link between justice perceptions and team learning, team leaders should actively promote justice-related behaviors within their teams. This includes addressing issues related to distributive, procedural, interpersonal, and informational justice. Team leaders should encourage team members to voice their concerns, provide them with opportunities to contribute to decision-making, and ensure that their decisions are based on unbiased information. Fostering and upholding perceptions of justice can enhance the teamwork environment, promoting both harmony and a culture of continuous learning within the team. Finally, organizations should establish clear policies and guidelines that promote fairness and justice in decision-making processes (Greenberg, 2011). These policies can serve as a foundation for leaders and team members to ensure that justice principles are upheld. In summary, this study underscores the significance of participative leadership, justice perceptions, and team learning in the workplace. Team leaders and organizations that prioritize these factors can create a conducive environment for continuous learning and growth, leading to enhanced team performance and a competitive advantage in an ever-changing business landscape.

### 5.4 Future research

This study contributes by identifying leaders’ induced justice perceptions among team members as playing a role in the relation between leadership behavior and team learning. Justice perceptions are conceptually different from other mediators previously identified, such as team behavioral integration (Carmeli et al., 2011; Chiu et al., 2021), psychological safety (Edmondson, 2012), and team trust (Carmeli et al., 2012). Therefore, it is necessary to better understand how leaders’ induced justice perceptions interact with other mediators to create the conditions that facilitate team learning. This would aid in producing a more accurate representation of the relationships among mediation variables, offering better insights into the process. Additionally, future research should study how the relationships between the mediators themselves can vary depending on the specific context and the dynamics within the team; for example, how team development factors that facilitate team learning, such as team composition (Chiu et al., 2021), stages of team development (Lorinkova et al., 2013), and the dynamics and evolving process of how teams learn over time (Wiese and Burke, 2019), are influenced by the fairness with which team leaders treat team members.

Finally, there is a need for more research in understanding how cross-country cultural differences might impinge on the necessary conditions to facilitate team learning. Supervisor’s fairness has been shown to be very relevant in high power-distance cultures where paternalistic leadership styles are prevalent because supervisor’s fairness can produce the difference between a benevolent paternalistic leader versus and a malevolent paternalistic leader, which becomes relevant in determining employees’ behavioral outcomes and beliefs (Bedi, 2020). Hence, future studies in differing cultural settings (e.g., varying in the power-distance culture trait), will allow to propose culturally appropriate interventions to promote team learning, which can help firms decrease the competitive gaps they might have with teams operating in other cultural settings where it might be easier to enable more team learning (Yorks and Sauquet, 2003).

## 6 Conclusion

The present study finds that team leader induced justice perceptions among team members play a mediation role in the influence of participative leadership behaviors on team learning. The mediation effect is a partial mediation, indicating that other mediators coexist, as well as a direct relation of participative leadership behaviors on team learning. Confirmatory factor analysis supports a four-factor model of leader induced justice perceptions (distributive, procedural, treatment and informational). The mediation model has a better fit to data if a second order factor is included which acknowledges partial shared variance across justice dimensions, suggesting the coexistence of justice dimensions specific variance and a ‘general justice’ factor. Finally, leader induced justice perceptions of team members required data aggregation at a team level, suggesting that they should be conceptualized as a team-level justice climate phenomenon.

## Data availability statement

The raw data supporting the conclusions of this article will be made available by the authors, without undue reservation.

## Ethics statement

The studies involving humans were approved by the Comité Ético Científico de Ciencias Sociales, Artes y Humanidades. Pontifica Universidad Católica de Chile. The studies were conducted in accordance with the local legislation and institutional requirements. The participants provided their written informed consent to participate in this study.

## Author contributions

AR: research design, data collection, data analyses, and complete writing.
